# Coagulation Abnormalities in Liver Cirrhosis: Diagnostic and Therapeutic Approaches

**DOI:** 10.3390/medicina62010104

**Published:** 2026-01-02

**Authors:** Dorotea Bozic, Ana Babic, Ivna Olic, Milos Lalovac, Maja Mijic, Anita Madir, Kristian Podrug, Antonio Mestrovic

**Affiliations:** 1 Department of Gastroenterology, University Hospital Split, Spinčićeva 1, 21 000 Split, Croatia; dora.bozic@hotmail.com (D.B.); ivna1211@gmail.com (I.O.); antonio.mestrovic1@gmail.com (A.M.); 2 Department of Gastroenterology and Hepatology, Clinical Hospital Merkur, Zajčeva 19, 10 000 Zagreb, Croatia; m_lalovac@net.hr (M.L.); mijic.maja@gmail.com (M.M.); 3 Department of Gastroenterology, Hepatology and Clinical Nutrition, Clinical Hospital Dubrava, Avenija Gojka Šuška 6, 10 000 Zagreb, Croatia; anita.madir@gmail.com; 4 Private Clinic MedPoint, Ulica Petra Krešimira IV 104, 21 210 Solin, Croatia; kpodrug@gmail.com

**Keywords:** liver cirrhosis, coagulopathy, bleeding, thrombosis, TEG

## Abstract

The liver is the primary site of synthesis for most coagulation factors and the central organ responsible for maintaining hemostatic equilibrium. In individuals with advanced liver disease, significant disruptions in coagulation homeostasis occur and consequently predispose patients to both thrombotic and bleeding complications. This review summarizes the pathophysiologic basics of liver cirrhosis-associated coagulopathies and discusses the diagnosis and treatment of common procoagulant conditions such as portal vein thrombosis and post-transplant hepatic artery thrombosis. The review also systematically addresses the most common bleeding complications, including spontaneous, portal hypertension-related, and periprocedural bleeding. The proper pre-procedural assessment of the bleeding risk is often required due to the great number of invasive procedures to which these patients are frequently subjected. The viscoelastic testing (thromboelastogram and thromboelastometry) seems to emerge as the most appropriate diagnostic method. Specific treatment recommendations for the correction of coagulation abnormalities and the management of severe thrombocytopenia are hereby presented.

## 1. Introduction

**Figure 1:**
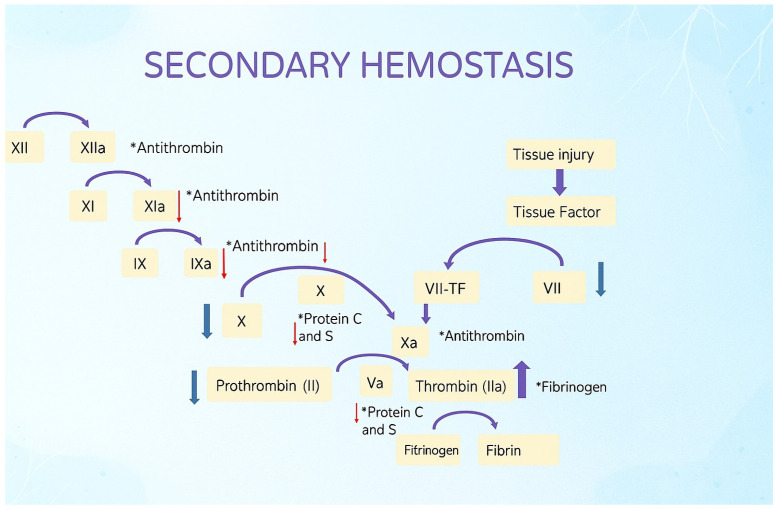
Coagulation cascade of secondary hemostasis. Blue arrows indicate anticoagulant activity, while red arrows indicate procoagulant activity. * Inhibitors are marked with an asterisk.

Blood coagulation is a tightly controlled process involving the endothelium, coagulation factors, platelets, and the fibrinolytic system. Under normal conditions, endothelial cells maintain an antithrombotic surface via thrombomodulin, nitric oxide, and prostacyclin, yet allow for rapid coagulation when vascular injury occurs. Primary hemostasis encompasses platelet adhesion and aggregation at the site of vascular injury, forming a temporary hemostatic plug, and secondary hemostasis involves the activation of the coagulation cascade (Figure 1), leading to fibrin clot formation and stabilization of the hemostatic plug [1]. The cascade is divided into intrinsic and extrinsic pathways, both of which converge on factor X activation, thrombin generation, and fibrin clot formation. Platelets adhere to exposed subendothelial collagen through von Willebrand factor (vWF), aggregate, and provide a phospholipid platform for the assembly of the coagulation complex [2,3,4]. In pathological conditions, disturbances in this delicate balance may lead to either hemorrhagic tendencies or thrombotic complications.

The liver is a central organ responsible for the hemostatic equilibrium, by being the primary site of synthesis for most coagulation factors, including fibrinogen and factors II, V, VII, IX, X, and XI, as well as natural anticoagulants such as protein C, protein S, and antithrombin. In individuals with advanced liver disease, significant disruptions in coagulation homeostasis occur and consequently predispose patients to both thrombotic and bleeding complications [5,6]. Liver cirrhosis is associated with intricate disturbances of the hemostatic system, involving a reduction in platelet count alongside elevated concentrations of vWF, decreased hepatic synthesis of procoagulant proteins and their physiological inhibitors, and significant alterations in fibrinolytic activity [7]. In cirrhosis, hepatocellular dysfunction leads to reduced production of procoagulant proteins, typically suggesting a bleeding predisposition [4,8]. However, recent evidence has challenged the traditional perception of cirrhosis as a purely hypocoagulable state. Studies show that while levels of procoagulant factors decrease, anticoagulant proteins decline proportionally or even to a greater extent [5,9,10]. Additionally, elevated levels of factor VIII and vWF, released in response to endothelial dysfunction and systemic inflammation, enhance platelet adhesion and thrombin generation. Concurrently, reduced levels of ADAMTS13 impair the cleavage of ultra-large vWF multimers, further contributing to a prothrombotic shift [11].

The main mechanisms responsible for thrombocytopenia in chronic liver disease are increased platelet destruction, splenic sequestration, and reduced hepatic synthesis of thrombopoietin (TPO), a key hormone that regulates megakaryocyte proliferation and platelet production [12]. In addition, bone marrow suppression and megakaryocyte dysfunction caused by chronic viral infections (particularly hepatitis B and C), alcohol consumption, iron overload, and drugs such as interferon further contribute to reduced platelet production [12,13]. Platelet destruction results from both immune-mediated and non-immune mechanisms. Reduced ADAMTS13 activity leads to the accumulation of vWF multimers, which promote platelet adhesion, aggregation, and premature consumption [12,14]. Infections and sepsis, frequent complications in advanced disease, further aggravate thrombocytopenia through systemic inflammation, activation of coagulation pathways, and enhanced platelet consumption [15]. Hypersplenism has long been recognized as a major contributor to thrombocytopenia in chronic liver disease (CLD) [16,17], although several additional pathophysiological mechanisms have since been identified. The development of portal hypertension leads to redistribution of splanchnic venous flow, resulting in splenic congestion and progressive splenomegaly that markedly increases platelet sequestration [18].

Moreover, alterations in the fibrinolytic system, including reduced synthesis of plasminogen and variable levels of tissue plasminogen activator (tPA), contribute to either hyperfibrinolysis or fibrinolytic shutdown, further complicating clinical assessment of coagulation status in cirrhosis [19]. Coagulation and fibrinolysis are also activated in the ascitic fluid of cirrhotic patients, with tissue factor-bearing extracellular vesicles driving local clotting and plasmin-mediated fibrinolysis potentially contributing to systemic fibrinolytic activity. These findings suggest that ascites not only reflects decompensation but also actively modulates hemostatic balance [20].

These pathophysiological changes contribute to what is currently described as a rebalanced yet highly unstable hemostatic state. As a result, even minor perturbations may shift this delicate equilibrium toward either a hypocoagulable profile, leading to an increased tendency for bleeding, or a hypercoagulable state, which predisposes patients to thrombotic complications [7,8].

## 2. Portal Vein Thrombosis in Liver Cirrhosis

Portal venous thrombosis (PVT) refers to an obstruction of the portal vein, its tributaries, or a combination of both. Acute PVT refers to a thrombus formed within the last 6 months, while chronic PVT lasts longer than 6 months [21]. It can be further classified, depending on the severity of the obstruction, as partial or complete.

The pathophysiology of PVT in the setting of cirrhosis is complex and likely arises from disruptions in one or more elements of Virchow’s triad, including reduced portal venous inflow, a hypercoagulable milieu, and localized injury to the portal vein endothelium [22]. Virchow’s triad describes the three primary factors predisposing to thrombosis: endothelial injury, stasis of blood flow, and hypercoagulability. Endothelial damage exposes subendothelial tissue, triggering platelet adhesion and coagulation. Stasis promotes local accumulation of activated clotting factors, while hypercoagulable states, whether inherited or acquired, shift the hemostatic balance toward thrombosis. In a prospective cohort study involving 310 patients with predominantly compensated cirrhosis observed over a median period of 48 months, Turon et al. reported that a portal vein flow velocity below 15 cm/s, rather than plasmatic indicators of hypercoagulability, was predictive of PVT development, indicating that blood stasis plays a dominant role in thrombogenesis [23]. Understanding these mechanisms is essential for identifying at-risk patients and guiding prophylactic strategies [24,25].

*Incidence*. PVT is a common complication of liver cirrhosis, with one- and three-year cumulative incidences of 4.78% and 9.34%, respectively, while the prevalence of PVT in cirrhosis is 13.92%. Of these, the incidence of complete PVT in cirrhosis reaches only 1.99%, while, conversely, in previously healthy individuals, complete thrombosis occurs more frequently [26]. Furthermore, both the incidence and prevalence are increased in patients with more advanced disease. Accordingly, a Child Turcotte Pugh (CTP) score of B or C, a higher MELD score, the presence of ascites, and a lower platelet count are predictors of PVT development [23,26,27,28,29,30,31]. On the other hand, there is no evidence supporting the role of inherent thrombophilia [23,28,32] or ABO blood type [33,34] in this condition.

*Diagnosis and imaging*. Patients with cirrhosis undergo routine ultrasound examinations as a part of screening for hepatocellular carcinoma (HCC); therefore, PVT is usually detected as an incidental finding. Ultrasound and Color Doppler (CD) provide a sensitivity and specificity of 89% and 92%, respectively, in the detection of PVT, which corresponds to the diagnostic accuracy of portography, but without the radiation exposure [35]. Multislice computed tomography angiography (MSCTA) is performed to assess the chronicity and extension of the thrombus [21,35,36]. The compression on extravascular structures is also evaluated. In patients with impaired renal function or iodine allergy, magnetic resonance angiography (MRA) is recommended since it offers the same diagnostic information [21,35]. When assessing the potential malignancy of the thrombus, contrast-enhanced ultrasound (CEUS) imposes itself as a method of choice. According to a meta-analysis published in February 2024 by Australian authors, which included 12 studies with a total of 712 patients, CEUS proved to be highly accurate in differentiating between benign and malignant causes of portal vein occlusion, with a sensitivity of 97.5% and a specificity of 98.1% [37]. This is due to neoangiogenesis and subsequent vascularization of the malignant thrombi, which leads to contrast imbibition, whereas benign thrombi are avascular and therefore do not exhibit imbibition [37].

*Management*. Spontaneous thrombus recanalization may occur in patients with partial PVT or following liver function improvement [38]. However, anticoagulant therapy (AC) is a cornerstone of treatment. A meta-analysis of 33 studies, including 1696 patients, revealed that the rate of portal vein recanalization increases following the initiation of anticoagulant therapy (RR = 2.61; 95% CI 1.99–3.43; *p* < 0.00001) [39]. A meta-analysis of 8 studies (353 patients), conducted by Italian authors, confirmed better recanalization rates when using AC. Authors found low-molecular-weight heparin (LMWH) to be more potent than warfarin (71% vs. 42%, respectively; *p* < 0.0001), although both were successful in slowing down the progression of PVT [40]. New research and studies [41,42,43], albeit limited, suggest that the use of direct oral anticoagulants (DOACs) promotes recanalization without increasing the risk of bleeding or mortality. Those findings, combined with their ease of use, beneficial pharmacokinetic and pharmacodynamic properties, and fewer drug interactions [44], make DOACs a go-to medication in cases of less severe PVT. All of the previously mentioned medications can be used in patients with the CTP score A and B. However, in patients with CTP score C and a high MELD score, LMWH is recommended due to its shorter half-life and the availability of a quick-acting reversal agent [36]. Additionally, studies show that mortality from any cause was significantly lower in patients who received AC compared to those who were left untreated, regardless of the extent of thrombosis or the degree of recanalization (HR 0.59; 95% CI 0.49–0.70) [45]. Finally, the PVT treatment should be individualized and based on liver function, bleeding risk, as well as the severity and chronicity of the thrombus. Certain authors consider that the chronic PVT does not require treatment, apart from the patients potentially undergoing orthotopic liver transplantation (OLT) [36].

All patients receiving AC should be assessed by computed tomography (CT) or magnetic resonance imaging (MRI) every 3 months to evaluate treatment response [36]. In patients without response or thrombosis progression, endovascular intervention should be considered [46]. Lack of response after 6 months of treatment significantly diminishes the probability of success, and it is recommended that the treatment be discontinued [47]. Since the recurrence rate of PVT after AC discontinuation is 38% in 2–5 months, patients who have had an adequate treatment response and are on the transplant list should continue using AC, while in all of the other cases, a therapeutic decision should be made on an individual basis [48,49,50].

Transjugular intrahepatic portosystemic shunting (TIPS) also plays a vital role in treating PVT. All cirrhotic patients with PVT and additional indications for TIPS, such as refractory ascites, hydrothorax, or esophageal variceal bleeding, should be considered for this procedure [7,51,52]. Studies also show that patients who were listed for a liver transplant and have undergone TIPS prior to liver transplant experience a high revascularization rate, improved post-transplant outcomes, and better overall survival [53,54].

PVT obstructs the portal vein, which can lead to exacerbation of already severe portal hypertension due to cirrhosis, so routine screening for esophageal varices (EGDS) is crucial [48]. The indications for primary and secondary prophylaxis of variceal bleeding remain consistent irrespective of the presence of PVT. Balancing the timing of anticoagulation therapy with endoscopic evaluation for varices is vital; however, studies have shown that the administration of AC does not increase the risk of variceal bleeding [40,45]. Notably, when performing endoscopic variceal ligation while on AC therapy, the additional risk factors remain minimal [55,56]. By facilitating recanalization, AC reduces portal pressure and paradoxically lowers the risk of bleeding [40]. Despite these findings, due to the scarcity of studies, differences persist in European and American guidelines on the optimal timing for AC administration [7,46].

*Prophylaxis*. The anticoagulant therapy is generally not recommended in patients with liver cirrhosis, but may be considered during hospitalization or immobilization. In recent years, statins are increasingly recognized for their pleiotropic effects in patients with liver cirrhosis, due to their anti-inflammatory and antiangiogenic properties, as well as modulation of fibrogenesis and hepatic endothelial function [57]. They display anticoagulant activity by suppressing tissue factor expression and enhancing endothelial thrombomodulin expression, thereby reducing thrombin generation. Moreover, statins interfere with fibrinogen cleavage, further limiting thrombin formation. Evidence from both experimental models and clinical studies indicates that statins also exert antiplatelet effects through early and late inhibition of platelet activation, adhesion, and aggregation [58].

These pathophysiological effects have fueled growing interest in the potential role of statins in the management of patients with liver cirrhosis. However, although they represent a promising therapeutic option in metabolic dysfunction-associated steatotic liver disease, with established benefits on cardiovascular outcomes and emerging evidence suggesting a potential role in reducing liver-related complications [59], more prospective randomized controlled trials are needed before widespread adoption of statins in the management of liver cirrhosis.

## 3. Hepatic Vein Thrombosis in Liver Cirrhosis

Hepatic vein thrombosis, also known as Budd-Chiari syndrome (BCS), is a rare, heterogeneous disorder characterized by the obstruction of hepatic venous outflow [60]. The obstruction can occur anywhere from small hepatic venules to the larger hepatic veins and inferior vena cava and its inflow into the right atrium [61]. BCS may occur in patients with liver cirrhosis as primary (due to prothrombotic state) or secondary (due to tumor-related vascular invasion or external compression, e.g., from HCC) [46,62,63,64]. Since primary BCS in liver cirrhosis is very rare, other risk factors such as thrombophilia, myeloproliferative neoplasms (MPN), paroxysmal nocturnal hemoglobinuria, antiphospholipid antibody syndrome, pregnancy, or oral contraceptives should be taken into consideration [46,64]. In addition, the JAK2V617F mutation, a common gain-of-function mutation leading to development of MPN, has been detected in considerable number of patients with PVT and BCS, and the screening for JAK2V617F should be performed as part of standard diagnostic work-up [46].

Furthermore, these two conditions are intertwined since liver cirrhosis may develop on a BCS basis. Namely, hepatic venous outflow obstruction causes an increase in intrasinusoidal pressure, leading to portal hypertension and liver congestion. This results in reduced hepatic perfusion, causing ischemic injury to hepatocytes, liver tissue fibrosis, and the formation of regenerative nodules, eventually progressing to liver cirrhosis [65,66].

*Incidence*. The incidence of BCS in patients with liver cirrhosis is extremely rare. A 2019 systematic review and meta-analysis of several high-quality studies from Europe and Asia found the pooled annual incidence of BCS to be 1 per million, and the pooled prevalence of 11 per million in the general population. To our knowledge, there are no specific data for patients with liver cirrhosis [64].

*Diagnosis and imaging*. Radiological methods for diagnosing BCS include Doppler ultrasound (DUS), computed tomography, and magnetic resonance imaging. A meta-analysis by Gupta et al. in 2020 assessed the sensitivity and specificity of these diagnostic methods, revealing sensitivity ranges of 89 to 93% [65].

DUS represents the first-line imaging modality for the diagnosis and follow-up of patients with Budd-Chiari syndrome due to its wide availability, cost-effectiveness, and lack of ionizing radiation. It allows for detailed assessment of the location and extent of venous obstruction, as well as the identification of intrahepatic veno-venous collaterals, a highly specific finding for BCS [67,68]. However, diminished visibility of hepatic veins or their altered flow, findings commonly seen in BCS, are not sensitive in patients with liver cirrhosis, since their hepatic veins are usually compressed by fibrotic parenchyma. The most common sign of BCS seen on CT is undetectable hepatic veins. Furthermore, CT is highly effective in detecting the indirect signs of BCS, such as hypoattenuating and heterogeneous hepatic parenchymal enhancement, perfusion abnormalities, and the presence of intrahepatic and extrahepatic collaterals. MRI is valuable in detecting all features of BCS; however, its use is dependent on availability and operator expertise [67].

*Management*. The primary objectives in the management of Budd-Chiari syndrome are the treatment of portal hypertension, the underlying thrombotic or malignant cause, and re-establishment of hepatic venous outflow [7]. The most widely accepted treatment paradigm, since it was first introduced in the early 2000s, is a stepwise therapeutic approach, depending on treatment response, which is determined by clinical and biochemical factors. Early and continuous anticoagulant therapy plays a key role in preventing thrombus progression and new clot formation; however, it is largely ineffective in achieving hepatic vein recanalization [69]. While low-molecular-weight heparin and vitamin K antagonists (VKAs) such as warfarin are most commonly used anticoagulants, some recent studies propose the use of direct oral anticoagulants [63]. The systematic review conducted by Wu et al. in 2025 suggests that DOACs are as effective and safe as traditional anticoagulants in the treatment of BCS [70]. Medical therapy alone does not produce a sustained clinical response in most patients, so additional interventional treatments are needed. Endovascular management is primarily based on anatomical recanalization and portosystemic shunt formation [71]. Since its introduction in 1993, TIPS has gained more significance in the management of BCS and nowadays is largely replacing surgical shunts, especially following the widespread adoption of PTFE-covered stents [69]. Liver transplantation (LT) is generally reserved for patients who do not respond to less invasive therapeutic modalities, but it may also be considered as a first-line treatment in cases of fulminant liver failure. In such acute settings, TIPS should be viewed as a bridging therapy, as it can stabilize critically ill patients and potentially eliminate the need for transplantation [7,72].

*Follow-up*. Hepatocellular adenomas and hepatocellular carcinoma may occur on the basis of BCS. The cumulative incidence of HCC in BCS has been reported at 6% over 7 years in the general population [7]. Imaging is vital in monitoring patients with BCS. Current guidelines recommend ultrasound and alpha-fetoprotein (AFP) measurement every 6 months, with additional evaluation using contrast-enhanced imaging and/or biopsy for indeterminate lesions [7,46].

## 4. Thrombosis in Patients After Liver Transplantation

Vascular complications of orthotopic liver transplantation represent some of the most serious and potentially life-threatening events affecting both patient and graft outcomes. Although their overall incidence is relatively low (7–13%), these complications significantly compromise graft viability by disturbing hepatic inflow or outflow. Among them, hepatic artery thrombosis (HAT) and portal vein thrombosis are the most frequent [73,74]. Table 1 summarizes the main vascular complications that can develop following OLT and highlights their shared risk factors with the hypercoagulable state and vascular alterations seen in liver cirrhosis.

### 4.1. Hepatic Artery Thrombosis

HAT represents more than 50% of all arterial complications after OLT [73]. HAT occurs in roughly 2–12% of adult liver transplants, with early HAT (within 30 days after OLT) being more frequent and life-threatening than late forms [73,75]. In a systematic review by Bekker et al., the overall incidence of early HAT was 4.4%, with adult rates around 2.9% [76]. HAT usually arises from technical, anatomical, or hemodynamic issues at the arterial anastomosis. Major risk factors include arterial kinking, small vessel caliber, size mismatch between donor and recipient arteries, prolonged cold ischemia time, and endothelial injury during procurement or implantation [73,76,77]. Specific surgical factors—such as arterial reconstruction, redo anastomosis, or use of arterial conduits—significantly increase the risk of thrombosis [75]. Recipient-related factors such as cryptogenic cirrhosis, prothrombotic states (e.g., antiphospholipid syndrome), and prior transarterial chemoembolization may further predispose to thrombosis [73]. Early HAT often presents as sudden graft dysfunction, biliary leakage, fever, or sepsis. In severe cases, it leads to acute hepatic necrosis and graft failure [75]. Conversely, late HAT may be asymptomatic or present with biliary ischemic complications such as strictures or abscesses. Laboratory abnormalities include rising transaminases, cholestasis, and coagulopathy. Because clinical findings are often nonspecific, early imaging surveillance is crucial for timely diagnosis.

*Diagnosis and imaging*. Doppler ultrasonography remains the cornerstone of both intraoperative and postoperative vascular monitoring after OLT [78]. It is a non-invasive, real-time, and highly sensitive modality for assessing graft perfusion. A normal hepatic artery waveform demonstrates a sharp systolic upstroke and continuous diastolic flow with a resistive index (RI) between 0.55 and 0.80. Absence of flow, no color signal or flat waveform, or a sudden drop of RI (<0.50) strongly suggest arterial occlusion [78,79]. Intraoperative ultrasound can detect anastomotic complications immediately, allowing for prompt revision before closure. Postoperatively, routine DUS is recommended daily during the first week and periodically thereafter. Postoperative surveillance protocols vary among transplant centers. When DUS findings are equivocal, contrast-enhanced CT angiography or MR angiography can provide confirmatory information.

*Management*. The cornerstone of treatment is rapid revascularization. Options include surgical revision, thrombectomy, or creation of an aorto-hepatic conduit [75]. Endovascular thrombolysis and angioplasty represent less invasive alternatives when performed early [73]. Retransplantation remains the only option for extensive infarction or graft necrosis, required in up to 50% of early HAT cases [75]. Preventive strategies focus on meticulous surgical technique, adequate arterial flow, and early postoperative surveillance. Antiplatelet prophylaxis has recently gained importance. Minciuna et al. showed that low-dose acetylsalicylic acid significantly reduced early HAT incidence in high-risk patients (3.2% vs. 21.3%) without raising bleeding risk [80]. In the absence of a universally accepted protocol, the decision to initiate antiplatelet therapy after liver transplantation is made at the discretion of individual transplant centers.

*Prognosis*. Outcome is determined by the promptness of diagnosis and the effectiveness of revascularization. Without prompt intervention, graft failure and mortality are common due to ischemic cholangiopathy and hepatic necrosis. Advances in Doppler surveillance, endovascular therapy, and surgical reconstruction have improved graft survival and reduced retransplantation rates [73,75,81].

### 4.2. Portal Vein Thrombosis

Portal vein thrombosis after OLT is relatively uncommon, with a reported incidence of 1–3% in adult recipients and slightly higher in living donor liver transplantation (LDLT) due to smaller vascular caliber and complex anastomoses [73]. Predisposing factors include technical issues during anastomosis, pre-existing portal vein pathology such as cavernous transformation, mismatch in vessel diameter, hypercoagulable states, and prolonged cold ischemia [82]. Additionally, intraoperative factors such as inadequate portal flow, venous kinking, or compression by perivascular hematoma contribute to thrombosis formation [73]. Systemic risk factors include the persistence of a prothrombotic state secondary to cirrhosis, endothelial dysfunction, and perioperative coagulation imbalance [82].

The clinical manifestations of PVT vary depending on the timing of onset. Early PVT (within 30 days after OLT) often presents with graft dysfunction, elevated transaminases, coagulopathy, and sometimes multi-organ failure. Late PVT may present with portal hypertension, ascites, splenomegaly, or variceal bleeding, typically with preserved graft function. However, many cases remain clinically silent, underscoring the importance of routine imaging surveillance.

*Diagnosis and imaging*. DUS remains the cornerstone for both intraoperative and postoperative vascular monitoring. It is a non-invasive, real-time tool that allows early detection of vascular complications. Normal portal venous flow is hepatopetal, continuous, and low-velocity (12–30 cm/s) [78]. Findings suggestive of PVT include absence of flow, echogenic thrombus, or reversal of flow direction (hepatofugal flow). Cavernous transformation may appear in chronic thrombosis [78]. When DUS findings are inconclusive, contrast-enhanced ultrasound, CT portography, or MR angiography may confirm the diagnosis and delineate the extent of thrombus. Routine postoperative ultrasound is recommended daily during the first week, weekly during the first month, and periodically thereafter, as early detection of altered hemodynamics allows timely management [78].

*Management*. Therapeutic strategies for PVT after LT depend on the timing and extent of thrombosis. Early PVT may be managed by surgical thrombectomy, re-anastomosis, or percutaneous thrombolysis and angioplasty, which can restore flow and prevent graft loss if performed promptly [73,82]. In partial or late PVT, anticoagulant therapy remains the mainstay. Low-molecular-weight heparin followed by oral anticoagulants such as warfarin or DOACs is commonly used, aiming to prevent thrombus progression and promote recanalization [78,83,84,85,86]. For acute, non-occlusive PVT, at least 6 months of anticoagulation is recommended, with Doppler follow-up to confirm recanalization. If complete or partial thrombosis persists, treatment can be extended up to 12 months, or longer in cases of chronic thrombosis or recurrent events. Once imaging confirms full recanalization and the patient remains asymptomatic, therapy may be discontinued, provided no underlying hypercoagulable condition exists [84]. Endovascular interventions, including stent placement or TIPS, are valuable alternatives in selected cases, particularly for recurrent or chronic thrombosis associated with portal hypertension [73]. In cases of complete occlusion with irreversible graft dysfunction, retransplantation may be the only curative option.

Given the high morbidity associated with PVT, preventive strategies are essential. Patients undergoing OLT with pre-existing PVT are at an inherently increased risk of early postoperative re-thrombosis due to altered portal hemodynamics, endothelial injury, and the prothrombotic state associated with advanced liver disease. Most transplant centers initiate early postoperative thromboprophylaxis using LMWH as soon as adequate hemostasis is confirmed. Transition to an oral anticoagulant (typically a vitamin K antagonist such as warfarin) is recommended once liver function and coagulation parameters stabilize. The duration of prophylaxis should be tailored according to the extent of thrombosis and the quality of portal vein reconstruction. Current evidence supports maintaining anticoagulant therapy for a minimum of 3 to 6 months after OLT, provided there are no bleeding complications. This approach aims to prevent recurrent thrombosis during the endothelial healing phase of the anastomosis [87]. According to Kirchner et al., anticoagulation for at least 3 months after OLT in patients transplanted with PVT significantly reduces early re-thrombosis rates without increasing major bleeding risk, provided that careful monitoring and early Doppler surveillance are maintained [87]. Table 2 summarizes the currently available DOACs with specific considerations related to their and warfarin use after liver transplantation.

*Prognosis*. The prognosis of PVT after OLT depends primarily on timely diagnosis and revascularization success. Early intervention may prevent graft loss, whereas delayed detection often leads to chronic portal hypertension and graft dysfunction [73,77].

## 5. Bleeding in Liver Cirrhosis

In general, bleeding in patients with liver cirrhosis can be divided into three main groups: bleeding related to portal hypertension, bleeding due to mechanical trauma or injury, and spontaneous hemostasis-related bleeding [89,90].

### 5.1. Spontaneous Hemostasis-Related Bleeding

Spontaneous hemostasis-related bleeding in cirrhosis is defined as an unprovoked hemorrhagic event of unexplained cause, divided into major and non-major bleeding, according to its severity, as proposed by the International Society on Thrombosis and Hemostasis [89,90]. Major spontaneous bleedings related to cirrhosis are as follows: massive spontaneous deep hematomas, spontaneous intracranial hemorrhage, spontaneous hematoperitoneum, orbital hemorrhage [91]. Less severe, non-major bleedings could be skin hemorrhages (bruises, petechiae, ecchymoses), mucosal bleeding (gum bleeding, epistaxis, menometrorrhagia), and dental root bleeding [91].

The association between liver cirrhosis and a predisposition to spontaneous intracranial hemorrhage has been previously demonstrated in numerous studies [92,93,94,95]. Although liver cirrhosis is generally associated with a higher incidence of stroke, there is a notable increase in the incidence of hemorrhagic stroke compared to ischemic stroke [95,96]. The estimated incidence of stroke per year in patients with liver cirrhosis is 2.17%, compared with 1.11% in patients without cirrhosis [96]. If we consider etiology, the likelihood of intracranial hemorrhage (ICH) is higher in alcoholic cirrhosis [94]. Moreover, in a Danish population-based case–control study, Grønbaek et al. have shown an increased incidence of intracranial hemorrhage even in patients with non-cirrhotic alcoholic liver disease. In the same study authors found an increased risk of ICH for patients with alcoholic liver cirrhosis (adjusted OR = 4.8, 95% CI: 2.7–8.3), non-alcoholic liver cirrhosis (adjusted OR = 7.7, 95% CI: 2.0–28.9) and non-cirrhotic alcoholic liver disease (adjusted OR = 5.4, 95% CI: 3.1–9.5), with women and those younger than 70 having the highest risk [94]. Some authors have attempted to objectify groups of patients with liver cirrhosis who are at higher risk of bleeding. In a recently published study, on 452,994 participants (mean age 57 years, 54% women), Parikh et al. showed that FIB-4 index > 2.67 was associated with an increased risk of hemorrhagic stroke (HR, 2.0; 95% CI, 1.6–2.6), highlighting a possibly predictive marker for determination of patients with higher risk of bleeding [97].

Falls are an important, but possibly preventable, cause of morbidity and mortality in cirrhosis [98]. In a retrospective study, Román et al. analyzed fall incidence in outpatients with cirrhosis and minimal hepatic encephalopathy (MHE). They have concluded that in one year, the incidence of falls was 40% in those with MHE compared with 13% in those without (*p* < 0.001) [99]. Results suggest the need for consideration of preventive therapy for hepatic encephalopathy in those with cognitive dysfunction [100]. Furthermore, Tapper et al. conducted a prospective study that enrolled 299 patients with compensated Child A and B (70% Child A) cirrhosis, without a prior episode of hepatic encephalopathy. Study showed that 1- and 3-year risk of falls was 29% and 50%, with 9% and 16% for injurious falls, meaning that 1 of 6 patients will experience injury during a fall over a three-year period. More than a two-fold increase in mortality was noticed in patients with falls [100]. Mentioned risk factors emphasize those subgroups that would most likely benefit from preventive interventions. In a previous study, Tapper et al. identified a predictive model for falls—FallSSS (Fall history, sodium, SF-8, and chair-stands), with past history of falls being the most potent predictor of falls [100].

Studies have unequivocally shown poorer treatment outcomes and increased mortality in the case of traumatic brain injury in patients with liver cirrhosis. According to the data available from the National Trauma Databank of America, patients with liver cirrhosis and brain trauma had longer need for mechanical ventilation compared to non-cirrhotic patients (2.9 ± 6.4 d vs. 2.0 ± 6.4 d, *p* < 0.001), with mortality rate almost twice higher (34.0% vs. 18.1%, OR = 2.34, 95% CI: 1.05–5.20, *p* = 0.035) [101]. In a similar study, authors concluded that among inpatients with cirrhosis, falls were frequent in those receiving benzodiazepines (51% vs. 17%; *p* < 0.0001) and antipsychotics (31% vs. 7.3%; *p* < 0.0001), urging caution while prescribing those medications [102]. In a longitudinal cohort study on 7296 patients with traumatic brain injury (2432 patients with liver cirrhosis and 4864 patients without) from National Health Insurance Research Database in Taiwan, authors concluded that patients with liver cirrhosis had a higher 1-year mortality (52.18% vs. 30.61%) and a 1.75-fold increased risk of mortality (95% CI 1.61–1.90) compared with noncirrhotic patients [103].

### 5.2. Role of Possible Prophylactic Therapy in Spontaneous Bleeding

As demonstrated, patients with liver cirrhosis are clearly susceptible to spontaneous bleeding. Current knowledge does not show benefits of using blood products or factor concentrates in preventing spontaneous hemostasis-related bleeding in patients with liver cirrhosis and abnormal standard coagulation parameters (INR, aPTT, fibrinogen, platelet count), highlighting the importance of conducting large observational studies that will first assess the frequency of spontaneous bleeding and its impact on mortality and clinical course in patients with cirrhosis, and thus the possible benefit of eventual coagulation status correction [90]. Regarding the stance for an active approach in spontaneous hemostasis bleeding, it is worth repeating that pro-hemostatic therapy is not the first-line treatment for bleeding in patients with cirrhosis [89,90]. This is strengthened by the fact that bleeding in liver cirrhosis is mainly not hemostasis-related, and that hemostasis is maintained even in severely ill patients. However, in the case of prolonged spontaneous bleeding, assessment of the underlying etiology and thus handling potential contributing factors, such as renal failure, infection, or sepsis, may reduce bleeding, while correction of hemostatic abnormalities can be considered on a case-by-case basis [104]. In those cases, point-of-care tools such as a viscoelastic test may be helpful in the assessment of candidates for a proactive approach [89]. A summary of the current position regarding the possible prophylaxis of spontaneous bleeding is shown in Table 3.

Although fibrinogen level is associated with a lower survival rate in decompensated cirrhosis and gastrointestinal bleeding, there is no clear evidence that prophylactic usage of complex containing fibrinogen has any effect on bleeding outcome [112,117]. Furthermore, there is no consensus on the appropriate threshold values for prophylactic platelet transfusions in cirrhotic patients or whether they should be given. In patients with thrombocytopenia without liver disease, preventive platelet transfusions are recommended in the case of a platelet count of 10 × 10^9^/L [118]. However, results from the prospective PRO-LIVER study on 280 cirrhotic patients (47% Child-Pugh B and C) followed up for about 4 years, concluded that platelet count does not predict unprovoked major or minor bleeding in cirrhotic patients [110].

### 5.3. Portal Hypertension-Related Bleeding

The first and most important example of bleeding related to portal hypertension is variceal bleeding. At the moment of diagnosis, nearly one-third of patients with cirrhosis have varices, with a 1-year rate of first variceal bleeding of 15% for large varices [119]. Incidence of variceal bleeding is related to the degree of portal hypertension, with hepatic venous pressure gradient (HVPG) > 20 mmHg being a predictor of treatment failure in patients with acute variceal bleeding [120]. The prevalence of esophageal varices is reported to be 42% in CTP grade A, 71% in CTP-B, and 76% in CTP-C cirrhosis [121]. Variceal bleeding is still one of the most frequent causes of death among cirrhotic patients, with a 15 to 20% mortality rate in the first 6 weeks following the index bleeding [90].

Importantly, hemostatic pathways have limited involvement in portal hypertensive bleeding [89]. Furthermore, studies concluded that bleeding outcomes are not worse in patients taking anticoagulant therapy [122]. Standard therapy for variceal bleeding includes band ligation, vasoactive therapy, and antibiotics in an acute setting, and beta-blockers for lowering portal pressure afterwards [90]. According to the latest EASL guidelines, correction of laboratory hemostatic abnormalities is not indicated [89]. However, in the case of failure to obtain hemostasis with standard endoscopic or medicament therapy, the decision to correct abnormalities in coagulation status should be made on an individual basis. In a meta-analysis of two controlled trials with 497 patients in total, Bendtsen et al. showed a beneficial effect of recombinant factor VIIa (rFVIIa) on the primary composite endpoint of control of acute bleeding, prevention of rebleeding day 1–5, and 5-day mortality in patients with advanced cirrhosis and active bleeding from esophageal varices at endoscopy. However, as the authors concluded, the negative side of the treatment is a potentially increased risk of arterial thrombo-embolic events (five thromboembolic events occurred in rFVIIa-treated patients compared to none in placebo-treated patients) [113]. This treatment might be considered in patients with a lack of control of bleeding after standard treatment. An international randomized, double-blind, placebo-controlled trial (HALT-IT) including 12,009 patients, analyzed the effect of a high-dose 24 h infusion of tranexamic acid on death and thromboembolic events in patients with acute gastrointestinal bleeding, of whom nearly half were with suspected variceal bleeding. The authors concluded that the use of tranexamic acid did not reduce death from gastrointestinal bleeding. Also, there was no beneficial effect in the subgroup analysis of patients with variceal bleeding. On the other side, venous thromboembolic events (deep vein thrombosis or pulmonary embolism) were higher in tranexamic acid group than in the placebo group (48 [0·8%] of 5952 vs. 26 [0·4%] of 5977; RR 1·85; 95% CI 1·15 to 2·98), with notice that higher risk was observed in group with variceal bleeding [116]. Restriction in red blood cell transfusion during variceal bleeding has been crucial in first-line management. It has been established that large volume overload directly increases portal pressure, with a higher rate of early rebleeding [123,124]. In an observational study by Mohanty et al., fresh frozen plasma transfusion in acute variceal bleeding was independently associated with poor clinical outcomes. Results showed that fresh frozen plasma (FFP) transfusion was associated with increased odds of mortality at 42 days (OR 9.41, 95% CI 3.71–23.90), failure to control bleeding at 5 days (OR 3.87, 95% CI 1.28–11.70), and prolonged hospitalization > 7 days (adjusted OR 1.88, 95% CI 1.03–3.42) [109]. Absence of benefit of FFP transfusion in the case of acute variceal bleeding proves its non-hemostatic nature [125].

In a case of active bleeding related to portal hypertension gastropathy, treatment should be aimed at lowering portal pressure, with vasoactive therapy in an acute setting and beta-blockers in a chronic setting [89,90]. An overview of current views regarding the possible usage of pro-hemostatic blood products and factors is presented in Table 3.

## 6. Invasive Procedures in Patients with Liver Cirrhosis

Clinicians are very often obliged to improve the coagulation status of patients with liver cirrhosis prior to various diagnostic or therapeutic procedures, such as abdominal paracentesis, liver biopsy, variceal band ligation, or local hepatocellular treatment. In order to reach appropriate decisions, the most important goal is to adequately evaluate their coagulation status and to properly characterize the invasiveness of the procedure.

According to the 7th International Coagulation in Liver Disease Conference held in 2019, low-risk procedures include abdominal paracentesis, thoracocentesis, dental extraction, upper and lower endoscopy, variceal band ligation, cardiac catheterization, and central venous placement [126]. Intermediate-risk procedures include lumbar puncture, percutaneous or transjugular liver biopsy, TIPS placement, PEG placement, sphincterotomy, as well as local HCC therapies (embolization, ablation), while the high-risk procedures include complicated endoscopical and all surgical procedures [126].

Janko N. et al. conducted a retrospective study that analyzed postprocedural bleeding rates in 566 procedures (17% high-risk and 83% low-risk) carried out in 233 patients with liver cirrhosis [127]. Overall, the bleeding occurred in 1.8% of procedures (0.4% in low-risk and 8.3% in high-risk groups). In patients with significant coagulopathy, which was defined as INR > 1.5 and/or platelets < 50 × 10^9^/L, the postprocedural bleeding rate was similar between the patients who did or did not receive blood products (3.1 and 1.9%, respectively). The only significant predictor of bleeding in the multivariate analysis was the high procedural bleeding risk category [127]. Similarly, in another cohort of patients with liver cirrhosis that underwent 60 low-risk procedures, no postprocedural bleeding was detected, both in patients with or without severe coagulopathy. However, in patients who underwent high-risk procedures, a bleeding rate of 17% was detected in patients with coagulopathy compared to 0% in patients without coagulopathy, although this difference was not statistically significant (*p* = 0.06) [128].

According to the EASL guidelines, there are no data supporting the use of FFP or platelet transfusion prior to abdominal paracentesis, and the guidelines recommend avoidance of LVP in the presence of disseminated intravascular coagulation [129]. However, the American Gastroenterological Association (AGA) emphasizes that patients with AKI or taking anticoagulants might be at an increased bleeding risk [130]. Raco J. et al. did not find an increased bleeding risk in the general population of patients taking anticoagulants that underwent abdominal paracentesis [131]. However, Kuperman et al. reported two cases of severe bleeding in patients taking apixaban with compensated cirrhosis following abdominal paracentesis. Further investigations are warranted to evaluate the safety of paracentesis in patients with liver cirrhosis taking NOACs [132].

Elective endoscopic variceal band ligation (EVL) is a frequently used method in primary and secondary prophylaxis of variceal bleeding. Drolz et al. retrospectively analyzed the incidence of UGIB after 787 elective EVLs and found the rate of 4.8%. Platelet count and fibrinogen levels showed no relation with the postprocedural bleeding, while the INR appeared to have an association, but only in the univariate analysis. More importantly, correction of the coagulation status did not improve postprocedural complication rates [133].

Lu et al. evaluated the risk of bleeding in patients with EV undergoing ERCP. They included 75 patients with LC in the analysis, among whom 45 had established EV (73% graded as high-risk EV). None of the patients had esophageal variceal bleeding, while one patient had an episode of bleeding from gastric varices [134]. Odewole et al. conducted an interesting study about bleeding complications from transesophageal echocardiography (TEE) in patients with LC and found the pooled incidence of bleeding of 0.37% (95% CI 0.04–0.94%) [135].

Blasi A. et al. conducted a multicenter retrospective study that evaluated the risk of major bleeding after percutaneous liver procedure (biopsy and ablation) in patients with (316) and without LC (1481). Among patients with LC, 14 (0.8%) experienced major bleeding, with 0.4% occurring during ablation techniques and 0.8% following the percutaneous liver biopsy. Only 24% (6/25) of patients with an INR > 1.5 were transfused with FFP, and 72% with Plts < 50,000 received platelet transfusion. Patients with LC were more frequently transfused (5.9% vs. 1.5%). Interestingly, none of the patients who met the criteria for transfusion experienced major bleeding, and this did not depend on the transfusion administration [136].

### Assessment and Treatment of Coagulopathy

Apart from the platelet count and fibrinogen values, PT (prothrombin time), INR (international normalized ratio), activated partial thromboplastin time (aPTT), and bleeding time (BT) are generally the most commonly used values to evaluate the bleeding risk. However, they are insufficient and even inaccurate for the adequate assessment of the coagulation status in patients with liver cirrhosis since they evaluate only the formation of small part of the thrombin quantity instead of the total amount [127]. Additionally, kidney injury and infection might also alter the bleeding risk [126].

On the other hand, viscoelasting methods that include thromboelastogram (TEG) or rotational thromboelastometry (ROTEM) are two widely available techniques that can compensate shortcomings of the generally used coagulation parameters. They graphically display the process of blood clot initiation, formation, strength, and lysis. These measurements are explained in the following paragraph, and have different names and reference values for two different techniques which also use distinct reagents (TEG and ROTEM). R time (TEG) and clotting time (CT, ROTEM) define the time needed to initiate clot formation. Alpha angle (TEG and ROTEM) describes the speed of clot formation (fibrin to fibrinogen conversion). K time (TEG) and clot formation time (CFT, ROTEM) measure the time needed to reach the 20 mm clot size. Maximum amplitude (MA) (TEG) and maximum clot firmness (MCF, ROTEM) measure the quality and stability of a clot. LY_30_ (TEG) and maximum lysis (ML, ROTEM) define the percentage of clot lysis 30 min and 60 min from the MA/MCF, respectively [137].

Each of these measurements speaks of the quality of the coagulation cascade (internal and external clotting factors), fibrinogen and platelet values, as well as the effect of anticoagulant/antiplatelet drugs, and may accordingly lead clinicians to proper correction of the coagulation status.

**Figure 2:**
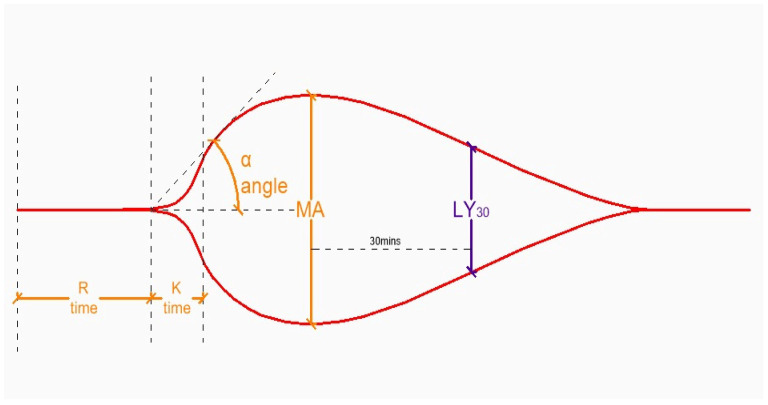
TEG/ROTEM graph. R (CT): clotting time; K (CFT): clot formation time; MA: maximum amplitude; LY_30_: clot lysis at 30 min.

Figure 2 and Table 4 depict the TEG/ROTEM graph with the parameter interpretation.

In recent years, the thrombin generation assay has emerged as a promising approach for assessing bleeding and thrombotic risk, as it evaluates the initiation, amplification, and inhibition of coagulation. However, the lack of assay standardization and well-defined cut-off values has limited its implementation in routine clinical practice [138,139].

Studies indicate that clinicians using TEG are still non-confident regarding its accuracy. Azer A. et al. published a retrospective study that included 89 patients with liver cirrhosis (277 TEG results). The analysis found that transfusion of blood products (FFP and platelets) was still indicated in patients with normal TEG values [140]. However, Shenoy et al. conducted a meta-analysis on the use of viscoelastic testing prior to non-surgical procedures in patients with LC. Included were 6 studies (367 patients) that compared viscoelastic testing to the standard of care (SOC), and the final analysis evaluated transfusion of blood products, as well as the postprocedural outcomes. Viscoelastic testing-guided transfusions resulted in a statistically significant decrease in the number of patients who received both FFP and Plts (SMD = −0.93 and SMD = −1.50, respectively). This transfusion decrease rate did not result in increased post-procedural bleeding (RR = 0.61, *p* = 0.09) or mortality (RR = 0.91, *p* = 0.93) [141]. Azer A. et al. conducted a review that included 40 papers, among which 22 found viscoelastic testing a better indicator of coagulation status than traditional tests, while 19 found that viscoelastic testing led to a reduction in blood product administration without increased hemorrhage or thrombotic risk [137].

In 2019, AGA published guidelines regarding the diagnosis and treatment of coagulopathies in liver cirrhosis and concluded that coagulopathy and thrombocytopenia do not require correction for **low-risk** procedures [130].

Table 5 presents specific treatment options for corrections of drug hypersaturation, deficits of clotting factors, thrombocytopenia, low fibrinogen levels, and increased fibrinolysis.

AGA proposed the following cut-off values requiring corrections before **high-risk** procedures: hematocrit < 25%, platelet count < 50,000, and fibrinogen < 120 mg/dL. According to AGA, raising the hematocrit value above the aforementioned threshold improves margination of platelets and hemostasis [130]. Fresh frozen plasma is no longer recommended for coagulopathy correction due to potential volume overload, an increase in portal pressure, and minimal effect on thrombin generation. Instead, application of the prothrombin complex concentrate is recommended due to the low-volume therapeutic effect. According to the 7th International Coagulation Conference, platelet transfusion is recommended prior to high-risk procedures or in cases of active bleeding in patients with platelets < 50,000/μL [126]. Since thrombocytopenia is often very difficult to correct with platelet administration only, due to splenic sequestration and short platelet survival, thrombopoietin agonists are a good alternative, although their effect requires time for proper correction (approximately 10 days) [130]. Finally, diminished fibrinogen levels may be corrected with cryoprecipitate or human fibrinogen concentrate, with the latter having the advantage due to low volume and no need for cross-matching [126].

## 7. Treatment of Severe Thrombocytopenia

Thrombocytopenia, defined as a platelet count below 150 × 10^9^/L, is the most prevalent hematological abnormality in chronic liver disease. Its prevalence ranges from 6% to 78%, increasing with disease progression and affecting up to 90% of patients with liver cirrhosis [12,13,142]. Mild (100–150 × 10^9^/L) and moderate (50–100 × 10^9^/L) thrombocytopenia are typically asymptomatic and have minimal clinical impact [12]. In contrast, severe thrombocytopenia (<50 × 10^9^/L) significantly increases the risk of both spontaneous bleeding and procedure-related hemorrhage, which is a major concern due to the frequent need for invasive procedures such as liver biopsy, endoscopy, or surgery in advanced chronic liver disease [12,143].

Management of thrombocytopenia in CLD focuses on ensuring procedural safety, minimizing bleeding risk, and reducing the need for platelet transfusions. Current strategies encompass supportive measures, interventional procedures, and pharmacologic therapies, with thrombopoietin receptor agonists representing a valuable option for selected patients to optimize platelet counts and decrease transfusion requirements prior to high-risk interventions [12].

### 7.1. Platelet Transfusion

The gold standard for managing thrombocytopenia in liver cirrhosis has traditionally been platelet transfusion, particularly before invasive procedures [142]. Despite the absence of universally defined thresholds, most contemporary guidelines advise platelet transfusion when counts fall below 50 × 10^9^/L for procedures associated with a moderate or high risk of bleeding [7,144]. For low-risk interventions such as paracentesis or diagnostic endoscopy, platelet counts > 20 × 10^9^/L are generally considered sufficient, while >100 × 10^9^/L is advised for neurosurgical or cardiac operations [145]. The benefit of platelet transfusion is transient, reflecting the short platelet lifespan (~72 h) and rapid splenic sequestration [12,146]. Repeated transfusions may result in alloimmunization, infection, transfusion reactions, and platelet refractoriness, while limited donor availability, cost, and the requirement for hospitalization further limit their routine use. Emerging evidence suggests that bleeding risk in cirrhosis does not correlate directly with platelet count, supporting a selective rather than prophylactic transfusion approach [12,142].

### 7.2. Interventional Management

(a) Splenectomy

Laparoscopic splenectomy can effectively and permanently correct hypersplenism and thrombocytopenia. However, its invasiveness and the risk of perioperative complications limit its use to selected patients, such as those with hypersplenism refractory to medical therapy or with concomitant hepatocellular carcinoma requiring curative resection [147,148]. Historically, open splenectomy was used to manage thrombocytopenia but was associated with high rates of bleeding and hepatic decompensation, leading to a preference for laparoscopic or shunt procedures [147,148]. Even with minimally invasive techniques, complication rates remain significant (2.5–17%), with portal or splenic vein thrombosis occurring in about 10% of cases [149]. Therefore, splenectomy is currently reserved for specific, carefully selected cases and is rarely performed in routine clinical practice.

(b) Partial splenic embolization (PSE)

Partial splenic embolization (PSE) is a minimally invasive alternative to splenectomy that selectively embolizes 50–70% of the spleen to reduce sequestration and raise platelet counts [142]. It provides sustained improvements in platelet and leukocyte levels in many patients with cirrhotic hypersplenism but carries risks such as post-embolization syndrome, splenic abscess, and portal vein thrombosis [150]. The platelet response and complication rate both increase with the extent of embolization, so limiting the treated volume is essential for safety. Hematologic benefits may last for months to years, though revascularization can cause recurrent thrombocytopenia. PSE is generally contraindicated in advanced decompensated cirrhosis (Child-Pugh C) [12,150].

(c) Radiofrequency ablation of the spleen

Radiofrequency ablation (RFA) of the spleen is a minimally invasive and cost-effective approach that has shown promising results in patients with cirrhosis and severe thrombocytopenia [151]. It carries a lower complication rate compared to other invasive procedures, though risks of hemorrhagic shock and intra-abdominal bleeding remain [152]. Further clinical studies with longer follow-up are needed to confirm its efficacy and safety.

(d) Transjugular intrahepatic portosystemic shunt and other shunt procedures

Shunt procedures, including portocaval, splenorenal, and transjugular intrahepatic portosystemic shunts, have been explored to reduce splenic congestion and platelet sequestration in chronic liver disease. By decompressing the portal and splenic circulation, TIPS effectively decreases portal pressure and may transiently improve thrombocytopenia, though without consistent long-term benefit [151]. Due to risks such as hepatic encephalopathy and shunt dysfunction, these procedures are reserved for specific indications, mainly refractory variceal bleeding or ascites, rather than correction of thrombocytopenia [153].

### 7.3. Pharmacological Therapies

Advances in understanding the pivotal role of thrombopoietin in platelet production have led to the development of TPO receptor agonists (TPO-RAs), which stimulate thrombopoiesis by activating the c-MPL receptor on megakaryocytes, thereby mimicking endogenous TPO activity [154].

(a) Thrombopoietin receptor agonists

The oral agents avatrombopag and lusutrombopag represent significant progress in the management of thrombocytopenia in cirrhotic patients requiring invasive procedures. Administered short-term (avatrombopag 40–60 mg once daily for 5 days; lusutrombopag 3 mg once daily for 7 days), these agents are initiated 1–2 weeks before the procedure to ensure an adequate rise in platelet count [12]. Pivotal phase III randomized controlled trials (ADAPT-1, ADAPT-2, L-PLUS-1, and L-PLUS-2) consistently demonstrated that both agents significantly increased platelet counts and reduced the need for platelet transfusions compared with placebo, without a significant increase in thromboembolic events [155,156]. Compared with platelet transfusion, TPO-RAs provide more predictable platelet responses, avoid transfusion-related risks, and simplify pre-procedural management. However, their use requires caution in patients with an elevated risk of thrombosis, such as those with portal vein thrombosis or advanced Child-Pugh C cirrhosis [157,158].

(b) Recombinant TPO and human cytokines

Recombinant human TPO and pegylated megakaryocyte growth and development factor initially showed efficacy in stimulating platelet production without major safety issues, but were discontinued due to the development of neutralizing antibodies [159,160]. Likewise, recombinant human cytokines, such as interleukin-1, approved for chemotherapy-induced thrombocytopenia, were limited by cardiovascular toxicity and flu-like adverse effects [161].

(c) Correction of underlying causes

Whenever possible, the underlying cause of liver disease should be identified and treated, as improving liver function can enhance endogenous thrombopoietin (TPO) production and gradually restore platelet levels. Targeted treatments such as antiviral therapy for hepatitis B or C, alcohol cessation [162], and metabolic control in non-alcoholic fatty liver disease support platelet recovery and help slow or reverse the progression of liver damage [12].

## 8. Conclusions

Liver cirrhosis is a very unstable hemostatic condition imagined as a scale that balances between procoagulant and anticoagulant states, in which minimal triggers can tip to one side and cause severe, even life-threatening complications. The task of a clinician is to be familiar with the assessment of the coagulation status of the patient, in which newer methods, such as viscoelastic testing, seem to have an advantage over other conventional methods. It is essential to recognize and treat complications of coagulation abnormalities in a timely manner, and to adequately prepare patients for invasive procedures to which they are frequently subjected, without causing additional harm and increasing the risks on the other side of the scale.

Additional research on the performance of viscoelastic testing and the effectiveness/harmfulness of certain drugs (such as NOACs) in patients with decompensated disease is required.

## Figures and Tables

**Table 1:** Vascular complications after liver transplantation and their association with pre-existing risk factors.

Complication	Typical Timing	Pathophysiology	Shared RF with Pre-LT Cirrhosis	Clinical Impact
Hepatic artery thrombosis (HAT)	Early (≤30 days) or late	Thrombosis at the anastomosis or due to endothelial injury; impaired arterial inflow to the graft and biliary tree	Hypercoagulable state, endothelial dysfunction, previous thrombosis, technical factors	Graft ischemia, biliary necrosis, graft loss, sepsis
Portal vein thrombosis (PVT)	Early or late	Thrombosis of the portal inflow due to sluggish flow, intimal injury, hypercoagulability	Portal hypertension, reduced portal flow, inherited/acquired thrombophilia	Impaired graft perfusion, portal hypertension, ascites
Hepatic vein thrombosis (HVT)	Early or late	Thrombosis or stenosis of hepatic venous outflow or anastomosis	Hypercoagulability, venous stasis, endothelial injury	Graft congestion, hepatomegaly, ascites, liver failure
Inferior vena cava (IVC) stenosis or thrombosis	Early or late	Anastomotic narrowing, compression	Venous stasis, surgical technical factors	Lower extremity edema, graft congestion

RF: risk factors; LT: liver transplantation; HAT: hepatic artery thrombosis; PVT: portal vein thrombosis; HVT: hepatic vein thrombosis; IVC: inferior vena cava.

**Table 2:** DOAC and warfarin-specific considerations after liver transplantation.

DOAC	Metabolism	Interaction Risk with IS	Preferred Use After LT	Clinical Recommendation
Dabigatran [84]	Prodrug, substrate of P-gp only	Strongly affected by Cys, increased exposure with Tac	Avoid in the early and unstable post-LT period	High risk of bleeding due to elevated plasma levels; avoid with CNIs or mTOR inhibitors
Rivaroxaban [85,86]	Metabolized via CYP3A4 (60%) and P-gp	Exposure increases with Tac and Cys	Use with caution in stable LT recipients	Monitor for bleeding; adjust dose or avoid concurrent strong CYP3A4/P-gp inhibitors
Apixaban [85,86]	Dual elimination via CYP3A4 (~25%) and P-gp	Least affected by CNIs; mild increase with Tac	Preferred DOAC in stable LT patients	Lower interaction potential; suitable for patients with preserved hepatic and renal function
Edoxaban [84]	Minimal CYP metabolism; P-gp substrate	Possible accumulation with Cys	Consider only with careful monitoring	Limited data post-LT; avoid with potent P-gp inhibitors
Warfarin [88]	CYP2C9, CYP1A2, CYP3A4	Multiple interactions but easily monitored via INR	Alternative during the early post-LT phase	Safe when close INR monitoring is feasible; unaffected by P-gp inhibition

DOAC: direct oral anticoagulant; IS: immunosuppressants; P-gp: P-glycoprotein; Cys: Cyclosporine; Tac: tacrolimus; CNI: calcineurin inhibitor; mTOR: mammalian target of rapamycin; CYP: cytochrome P450 enzyme; INR: international normalized ratio.

**Table 3:** Current views on administration of factor concentrates or blood products in the prevention of spontaneous bleeding (A) and in acute portal hypertension-related bleeding (B).

Factor Concentrates or Blood Products	Current Recommendations (A)	Special Notice (A)	Current Recommendations (B)	Special Notice (B)
Vitamin K	Not supported	No improvement of INR in liver cirrhosis [105,106] Not evaluated in the prevention of spontaneous bleeding	Not supported	No improvement of INR in liver cirrhosis [105,106] No reduction in rebleeding within 30 days in patients with cirrhosis and UGIB [107]
FFP transfusions	Not supported	Potential transfusion-related circulatory overload, transfusion-related acute lung injury [90,108]	Not supported	Potentially increases mortality, fails to control rebleeding [109]
Platelet transfusions	Not supported, controversial (low-grade evidence)	No clear-cut evidence suggesting a role in the prevention of spontaneous bleeding [90,110]	Not supported, controversial (low-grade evidence)	Potentially TEG-guided, no difference in rebleeding and 6-week mortality [111]
Cryoprecipitate (fibrinogen, factor VIII, factor XIII, and vWF)	Not supported	No effect on mortality risk or bleeding outcome [112]	Not supported yet, potential role	Benefit in acute bleeding, prevention of rebleeding on day 1–5, and 5-day mortality [113]
Thrombopoietin receptor agonists	Not supported	Possible role in the prevention of procedure-related bleeding [114]	Not supported	Possible role in the prevention of procedure-related bleeding [114]
Tranexamic acid	Not supported	Proved ineffective in subarachnoid hemorrhage [115] Possible thromboembolic adverse events [116]	Not supported	No benefit shown [116] Possible thromboembolic adverse events [116]

UGIB: upper gastrointestinal bleeding; FFP: fresh frozen plasma; TEG: thromboelastography; vWF: von Willebrand factor.

**Table 4:** Interpretation of TEG/ROTEM aberrancies [134].

TEG Parameter (ROTEM)	Aberrancy	Cause
R (CT)	↑	↓ clotting factors, drugs (heparin, warfarin, NOACs)
K (CFT)	↑	↓ fibrinogen ↓ fibrinogen, ↓ clotting factors, drugs
A (α)	↓	
MA (MCF)	↓	↓ platelets, ↓ fibrinogen
LY_30_ (ML)	↑	↑ fibrinolysis

TEG: thromboelastogram; ROTEM: rotational thromboelastometry; NOACs: novel oral anticoagulants.

**Table 5:** Specific treatment depending on the coagulopathy causes [126,130].

Cause	Treatment	Dose (Administration Route)
Heparin	Protamin sulphate	1 mg/100 IU heparin
Warfarin	Phytomenadion	0.5–1 mg (po/iv)
Apixaban, rivaroxaban	Andexanet α	480–1760 mg
Dabigatran	Idarucizumab	5 g
↓ Clotting factors	FFP	10–15 mL/kg (iv)
	PCC	25–30 IU/kg (iv)
↓ Platelets	Platelet transfusion	1 dose /10 kg BW (iv)
	Avatrombopag Lusutrombopag	40–60 mg/day over 5 d (po) 3 mg once daily over 7 d
↓ Fibrinogen	Cryoprecipitate	1 U/10 kg BW (iv)
	Fibrinogen concentrate	1–2 g (iv)
↑ Fibrinolysis	Tranexamic acid	500–1000 mg × 2–4/day (po/iv)

mg: milligram; IU: international unit; po: per os; iv: intravenous; g: gram; mL: milliliter; kg: kilogram; BW: body weight; d: days; PCC: prothrombin complex.

## Data Availability

No new data were created or analyzed in this study.

## Abbreviations

The following abbreviations are used in this manuscript:

TEG	Thromboelastogram
vWF	von Willebrand factor
TPO	Thrombopoietin
CLD	Chronic liver disease
tPA	Tissue plasminogen activator
PVT	Portal venous thrombosis
cm/s	Centimetre per second
CTP	Child Turcotte Pugh
HCC	Hepatocellular carcinoma
CD	Color Doppler
MSCTA	Multislice computed tomography angiography
MRA	Magnetic resonance angiography
CEUS	Contrast-enhanced ultrasound
AC	Anticoagulant therapy
RR	Risk ratio
CI	Confidence interval
*p*	*p*-value
LMWH	Low-molecular-weight heparin
DOACs	Direct oral anticoagulants
HR	Hazard ratio
OLT	Orthotopic liver transplantation
CT	Computed tomography
MRI	Magnetic resonance imaging
TIPS	Transjugular intrahepatic portosystemic shunt
EGDS	Esophagogastroduodenoscopy
BCS	Budd-Chiari syndrome
MPN	Myeloproliferative neoplasm
DUS	Doppler ultrasound
VKAs	Vitamin K antagonists
PTFE	Polytetrafluoroethylene
LT	Liver transplantation
AFP	Alpha-fetoprotein
HAT	Hepatic artery thrombosis
HVT	Hepatic vein thrombosis
IVC	Inferior vena cava
RI	Resistive index
LDLT	Living donor liver transplantation
P-gp	P-glycoprotein
CNIs	Calcineurin inhibitors
mTOR	Mammalian target of rapamycin
CYP	Cytochrome P450 enzyme
INR	International normalized ratio
ICH	Intracranial hemorrhage
OR	Odds ratio
FIB-4	Fibrosis-4 index
MHE	Minimal hepatic encephalopathy
d	Days
aPTT	Activated partial thromboplastin time
UGIB	Upper gastrointestinal bleeding
FFP	Fresh frozen plasma
HVPG	Hepatic venous pressure gradient
mmHg	Millimeters of mercury
EASL	European Association for the Study of Liver
rFVIIa	Recombinant factor VIIa
h	Hour
PEG	Percutaneous endoscopic gastrostomy
LVP	Large volume paracentesis
AGA	American Gastroenterological Association
AKI	Acute kidney injury
NOACs	Novel oral anticoagulants
EVL	Endoscopic variceal band ligation
EV	Esophageal varices
ERCP	Endoscopic retrograde cholangiopancreatography
TEE	Transesophageal echocardiography
LC	Liver cirrhosis
Plts	Platelets
PT	Prothrombin time
BT	Bleeding time
ROTEM	Rotational thromboelastometry
CT (ROTEM)	Clotting time
CFT	Clot formation time
mm	Millimeter
MA	Maximum amplitude
MCF	Maximum clot firmness
LY30	Clot lysis at 30 min
ML	Maximum lysis
PCC	Prothrombin complex
SOC	Standard of care
SMD	Standardized mean difference
mg	Milligram
IU	International unit
po	Per os
iv	Intravenous
g	Gram
mL	Milliliter
kg	Kilogram
BW	Body weight
mg/dL	Milligram per deciliter
µL	Microliter
L	Liter
PSE	Partial splenic embolization
RFA	Radiofrequency ablation
TPO-RAs	Thrombopoietin receptor agonists
